# Reflections on ‘Decolonizing’ Big Data in Global Health

**DOI:** 10.5334/aogh.3709

**Published:** 2022-07-21

**Authors:** Danya M. Qato

**Affiliations:** 1 University of Maryland Baltimore, School of Medicine & School of Pharmacy, US

**Keywords:** big data, health services research, global health, epidemiology

## Abstract

While much has been written about the role of ‘big data’ in health services research and epidemiology, there has been less exploration of the imperative of data sovereignty on informing the ethics of health services research and global health more broadly, especially in the context of decoloniality in an era of ‘big data.’ In this viewpoint, epidemiologist and health services researcher Qato offers a brief exploration of some questions that may drive this effort: is ‘decolonizing’ health data necessary? If so, what are the stakes, and who sets the terms? What would a decolonized data infrastructure necessary for health systems equity globally look like?

## Introduction

The topic of data sovereignty has seen a resurgence in the past decade. Among other complex underlying reasons, the expanding development and availability of large electronic health record and healthcare claims data; enhanced computational tools to analyze data; proliferation of high-speed and high-capacity data servers; and the ethical social and commercial concerns associated with the procurement, analysis, and storage of data have played a part in accelerating these debates. The fields of health services research and epidemiology in particular have been able to leverage electronic health record data and healthcare claims data to undertake varied and previously elusive observational studies. These studies can examine complex healthcare questions such as those related to the impact of policies on population health outcomes and the safety of medications. Colloquially, this resource has been referred to as ‘*big data*.’ The *data* here is any data that is ‘generated, created, collected and retained in any form or medium related to an individual patient, in, during, or part of a clinical or clinical research encounter [1].’ The *big* refers to the increased volume and breadth and depth of the data amassed, across ever-growing populations and over longer periods of time [2].

Another parallel phenomenon has been the uptake of the notion of *decoloniality* in global health discourse. The term ‘decolonizing’ has been ascribed to endeavors across the spectrum from educational syllabi re-construction to upending whole fields of inquiry. ‘Decolonizing’ global health discourse has been especially energized as the COVID-19 pandemic illuminated the widening chasm of healthcare inequities (related to mortality and access to the COVID-19 vaccine for example) between wealthier colonizing nation states and low-income states that were previously colonized or remain colonized [3,4]. That the tenor and locus of these conversations has largely continued to focus on colonizing or formerly colonizing nation state actors has been criticized for reifying the paternalistic, colonial, hierarchical structure, and power dynamics woven into the very fabric of the field of global health [5]. Epidemiologist Madhu Pai elaborates on the contours of this dynamic conversation and in doing so cites scholar Themrise Khan, who opines: ‘*The focus should instead be on the South, by the South itself. Not the South being a focus of the North. Southern countries need to trust each other more and come together as a coalition to support each other. There is no reason for us to continue looking to the West as the harbingers of prosperity*’ [6]. In this vein, several scholars have previously posed the million dollar question on the prospects of decolonizing the deeply colonial enterprise of ‘global health,’ asking whether the very process of decolonization of global health would usher in its demise [7,8]. These perspectives, along with the belief that ‘decolonization is not a metaphor’ [9] were foremost on my mind as I considered writing this piece.

The promise and perils of ‘big data’ analytics in shaping how we approach strategies to achieving health equity have been written about elsewhere [10]. There has been less exploration of the imperative of data sovereignty on informing the ethics of health services research and global health more broadly, especially in the context of decoloniality in an era of ‘big data’. This viewpoint will offer a brief and preliminary exploration of some questions that may drive this effort: is ‘decolonizing’ health data necessary? If so, what are the stakes, and who sets the terms? What would a decolonized data infrastructure necessary for health systems equity globally look like?

For the purposes of this brief, I rely on the following definition of data sovereignty that conceives of it as a ‘right’: Data sovereignty is the right of a nation to collect and manage its own data [11].While this definition emphasizes the particular rights of nations, especially indigenous peoples and nations [12], I deploy a rights-based definition that also sees patients, as individuals and as groups, as having a distinct and exceptional right to determine the parameters of the collection, utilization, accessibility, and use of their data. I am less interested here in the calls for patient ownership of data for purposes of monetization per se, but rather in their wresting power over and control of their data from private, profit-driven healthcare data corporations or non-native governmental and non-governmental organizations, who, save for a few exceptions, have been the key material beneficiaries and stewards of the big data enterprise on the global scale.

### On the imperative to decolonize health data

Many powerful international organizations, including the World Health Organization (WHO) [13] and the United States Agency for International Development (USAID) [14], have been invested in supporting the burgeoning electronic health records systems that ultimately funnel data to growing big data analytic firms (also referred to as electronic medical record or health information systems) of low and middle-income countries. On the face of it, if consistently and appropriately used, electronic health records or EMRs provide a longitudinal picture of the continuum of care for patients [15]. The World Health Organization (WHO) report on the Global Strategy for Digital Health outlines their vision for accelerated adoption of data analytics as such:

The vision of the global strategy is to improve health for everyone, everywhere by accelerating the development and adoption of appropriate, accessible, affordable, scalable and sustainable person centric digital health solutions to prevent, detect and respond to epidemics and pandemics, developing infrastructure and applications that enable countries to use health data to promote health and well-being, and to achieve the health-related Sustainable Development Goals and the triple billion targets of WHO’s Thirteenth General Programme of Work, 2019–2023.

While the healthcare benefits—namely related to improving continuity and quality of care and disease surveillance—of building an architecture and supporting infrastructure for data procurement, storage, and sharing within and across healthcare institutions are outlined in the WHO report, what is not addressed is the potential for intentional and unintentional misuse of data that may exacerbate and widen within country and between country health inequities and put particularly vulnerable patient populations in harm’s way. We also know from a vast array of research that racism and bias are woven into machine learning and data algorithms that are broadly deployed for healthcare decision-making and policy-shaping in the United States [16,17]. Often unbeknownst to them, these patient populations may be subject to ‘potentially stigmatizing, discriminating or exclusionary consequences [18].’

It is no stretch then to ask how emergent health data systems, in many cases funded by US and EU development and donor agencies [19], will be constructed and linked, and in whose hands they will fall? On the one hand, these questions gesture to the concept of data *ownership*, on the other, and for the purposes of this piece, they are gesturing to first and foremost to a notion of data *sovereignty*.

For example, while the MEASURE Evaluation program funded for the past two decades by USAID sought, ‘to help improve data collection, data quality, and the global capacity for research [20],’ it does not assert a claim of sovereignty (of any kind and in any formulation) or of power to the nations and populations in which they are based. Ultimately, those countries are beholden to the whims of the grant maker or donor agency (in this case USAID) who will determine, perhaps in consultation with the community or perhaps not, what methodologies will be used to construct and analyze data, what vendors will be contracted to house the data, and what data points and populations will be incorporated (or ignored) by design. Granted, there are community-based information systems as well as data demand and use programs that ostensibly center the populations being supported. Yet these programs do not explicitly address the myriad of questions and challenges that are plaguing better-financed health systems that are not reliant on international aid for the development of their data infrastructure, including the US. Perhaps this is by design. While intimations of accountability with respect to ensuring patient privacy and data quality are invoked, there is very little by way of tools for accountability and redress if ever the privacy of individual patients or patient groups is compromised. Additional salient questions especially centered on data governance remain unanswered: who ultimately controls stewardship of the data? Where will the data be stored and who and what entities will have access? For what purposes? Have safeguards been instituted to ensure appropriate use of the data to improve health outcomes and mitigate disparities? Who sets the terms for these safeguards and what quality control mechanisms have been built to ensure safeguards are actually working to prevent harm? Many of these conversations and debates remain compartmentalized. This fragmentation is in part due to the nature of developing context specific and responsive programs but also as a result of the multitude of gatekeepers at the helm, each with undoubtedly shared yet competing interests.

### What might a decolonized health data infrastructure look like

In order to envisage a decolonized health data infrastructure that supports health equity within and between formerly colonized and colonizing countries and populations, one must first envisage what a decolonized global health architecture would look like. Medical scholar Ijeoma Nnodim Opara, in the powerful piece entitled ‘It’s Time to Decolonize the Decolonization Movement’ [5], states that a key to a decolonizing mission is centering the knowledge of ‘indigenous and (neo) colonized decolonial and anticolonial liberation movement scholars, thinkers, strategists, and activists,’ upending of the power dynamic that drives and underlies most global health endeavors, as well as an emphasis on intersectionality and interdependence. In addition, Opara states that decolonizing the decolonization movement requires the work ‘be defined, led by, and the benefits reaped by *the Majority people of the world*.’ This anticolonial and revolutionary ethos should animate all aspects of building a decolonized data infrastructure as well. There are particularities to data infrastructure construction however, and some of these conditions are outlined as below:

1. *The community is always at the center*: In order to actualize a commitment to decolonizing health data at the community, national and international level, community-based boards of oversight that do not replicate the internal and external power dynamics that development agencies have historically relied on to undergird their power must be established. These oversight boards can serve as quality checks and balances systems to ensure equitable and responsible utilization and operation of data systems, as well as accountability systems if safeguards, such as patient privacy, are compromised. They can also ensure that the very concerns and concepts brought in to demarcate the parameters of the conversation do not reify Western ideas but rather reflect their very own values and concerns [21].
2. *Investing, entrusting, and centering a diversity of indigenous and local scholars, leaders, and scientists*: In developing and designing algorithms for healthcare, the knowledge and lived experiences of leaders, scientists, and scholars representing the diversity of the population from which data will be collected should be incorporated. Oftentimes, only a defined subset, typically upper-class members of the population, is represented in these leadership circles and is tokenized in an effort to project representation [6]. A central aim of decolonizing global health data should be to leverage the heterogeneous experience and knowledge base of the totality of the population represented, enabling greater understanding of populations and moving from crude representations of homogenous populations to granular understanding of the particularities and needs of particularly vulnerable subgroups.
3. *Build algorithmic transparency and accountability transparency*: Populations should have access to and an understanding of all aspects of the procurement, analysis, and storage of their data. Any one individual or group should be able to map the relationship between data vendors, and ultimately understand and retell the narrative that may be shaped based on these linkages in their data points. In other words, people should be aware of the story that is being told and sold about their health and their lives, and they should be at the helm of shaping and writing these narratives. As noted by the German Ethics Council: ‘Even if total control over one’s data trail is impossible in a digital society, people nonetheless consider it important that they be able to determine…how their data are used and reused [18].’ Transparency in this process also requires a reckoning with the digital divide within communities. Facilitating communications and access, where appropriate, to populations that are yet unable to access the internet, for example, are crucial to realizing full transparency and accountability.
4. *Consent, consent, consent*: If we are to understand sovereignty as a matter of rights and as a matter of power over one’s own story told through *big data*, then central to this story is consent. It goes without saying that if one’s data is to be used to construct policies that ultimately impact the very populations from which the data is derived, then those populations should consent to their data being deployed to help them or in some cases, weaponized against them. This is not a matter of training whole populations to serve as data programmers, but rather to create a quality narrative control system, whereby populations are continuously ‘brought in’ to register their agreement, dissent, or other misgivings about conclusions that shape policy and care provision happening as a result of their own data analyzed on their behalf. Only then can the limitations inherent to data science be addressed.
5. *Beyond abstraction*: As dialogue intensifies on the promise and pitfalls of big data in service of population health, there is a need to speak clearly and without equivocation about what researchers, advocates, and anticolonial scholars mean when we say ‘decolonize’ global health and health data infrastructures. Hummel et al., write of the multivaried and often vague renderings of ‘data sovereignty’ that are not intuitive and make shared understandings difficult. They note:
  Implicit or explicit contention, controversy, and negotiation processes about what data sovereignty means and should mean suggest that discussants seek to leverage the notion towards a variety of different ends. Yet, disputants might be talking past each other or make vague policy demands if they deploy the concept without being explicit about which of the various potential connotations are intended, and how the respective claim is supported [22].

It is past time advocates for a global health system rooted in equity and repair speak *to* each other rather than *past* each other. Such collective conversations enable the development of strategies that are not only more effective but more just, representative, and accountable. They also commit to a non-totalizing agenda that does not treat people as monoliths, but rather as individuals within communities each with their own sets of needs and perspectives.

In this brief viewpoint, I sought to preliminarily explore the primary questions and conditions that *may* help us map a decolonized global health data architecture whose construction is led by the very people who ‘populate the dataset.’ The benefits of any such approaches taken singularly, without a parallel effort to dismantle the power asymmetry and the colonial and capitalist hierarchies that foreclose the full actualization of health for the ‘wretched of the earth [23],’ will indeed be limited. What is clear is that we must move beyond platitudes and work towards solutions that force imperial powers and their corporate machinery out of the trade of selling in the sickness and health of populations and demand that they, and international aid agencies writ large, cede control and redistribute the wealth they have accumulated through colonialism (and other practices of exploitation) back to the patients, people, and populations whose stories are being told (and sold) through the accumulation of data.
